# Mathematical framework for human SLE Nephritis: disease dynamics and urine biomarkers

**DOI:** 10.1186/1742-4682-7-14

**Published:** 2010-05-17

**Authors:** Paula Budu-Grajdeanu, Richard C Schugart, Avner Friedman, Daniel J Birmingham, Brad H Rovin

**Affiliations:** 1 Mathematical Biosciences Institute, Ohio State University, Columbus OH 43210, USA; 2 Department of Mathematics, Western Kentucky University, Bowling Green KY 42101, USA; 3 Department of Mathematics, Ohio State University, Columbus OH 43210, USA; 4 Department of Internal Medicine, Division of Nephrology Ohio State University College of Medicine, Columbus OH 43210, USA

## Abstract

**Background:**

Although the prognosis for Lupus Nephritis (LN) has dramatically improved with aggressive immunosuppressive therapies, these drugs carry significant side effects. To improve the effectiveness of these drugs, biomarkers of renal flare cycle could be used to detect the onset, severity, and responsiveness of kidney relapses, and to modify therapy accordingly. However, LN is a complex disease and individual biomarkers have so far not been sufficient to accurately describe disease activity. It has been postulated that biomarkers would be more informative if integrated into a pathogenic-based model of LN.

**Results:**

This work is a first attempt to integrate human LN biomarkers data into a model of kidney inflammation. Our approach is based on a system of differential equations that capture, in a simplified way, the complexity of interactions underlying disease activity. Using this model, we have been able to fit clinical urine biomarkers data from individual patients and estimate patient-specific parameters to reproduce disease dynamics, and to better understand disease mechanisms. Furthermore, our simulations suggest that the model can be used to evaluate therapeutic strategies for individual patients, or a group of patients that share similar data patterns.

**Conclusions:**

We show that effective combination of clinical data and physiologically based mathematical modeling may provide a basis for more comprehensive modeling and improved clinical care for LN patients.

## Background

Autoimmune diseases occur when the immune system recognizes normal healthy tissues as foreign and attacks them. Systemic lupus erythematosus (SLE) is a chronic inflammatory autoimmune disorder that may affect the skin, joints, kidneys, and other organs. Lupus nephritis (LN) refers to the kidney disease caused by SLE. LN is associated with a worse prognosis than non-renal SLE [1,2], and can lead to chronic kidney disease (CKD). The pathogenesis of LN is complex and appears to be influenced by environmental and genetic factors [3]. Anti-DNA antibodies or immune complexes which contain these antibodies, are deposited in the kidney, which results in activation of the complement system, This leads to tissue inflammation and damage, and the consequent release of DNA, nuclear material, and cell debris. These products of tissue damage can serve as antigens, further stimulating the immune system and increasing the intrarenal inflammatory response. Clinical signs of LN include blood and protein in the urine, deterioration of kidney function, and high blood pressure. LN is typically characterized by exacerbations/relapses of disease activity (flares) and remissions (after treatment).

The accumulation of immune complexes in the renal glomeruli is pathogenic in LN, so there have been significant efforts directed toward developing treatments that control the formation, deposition, and clearance of immune complexes. Because there are multiple categories of lupus kidney disease, treatment is based largely on histologic severity [4,5]. The goal of treatment is to resolve the inflammation caused by the immune complexes and improve kidney function. Although the disease cannot be cured, aggressive immunosuppression is often effective in controlling renal flares. Despite improving disease outcome, these drugs are associated with significant morbidity and mortality.

Until more specific and less toxic therapies are developed, it is important to use the currently available immunosuppressive drugs more effectively and limit their toxicity. One way to improve current therapy is to monitor LN flare activity, accurately predict who will flare, when the flare will occur, and at what level of intensity, and plan the treatment accordingly, with the goals of forcing remission quickly, and minimizing cumulative immunosuppressive dose. Such effective approaches, however, are dependent on identifying biomarkers that monitor LN flare activity. Biomarkers discovery for SLE is an intense area of research [6-9]. Considerable efforts to validate biomarkers that best reflect flare status suggest that a panel of biomarkers rather than a single candidate will be needed. To determine which set of biomarkers is to be used will require the integration of biomaker data into a model of renal flare.

The present work presents a mathematical framework to correlate physiological processes relevant to LN with observed patient disease profiles. The differential equations model developed here is based on the dynamics of a few key components of the immune system and their effects on tissue damage. The complexity of the disease is effectively captured by this model, which qualitatively reproduces the clinical variations observed in LN patients undergoing therapy. Relevant parameter values are estimated using results of urine biomarker discovery studies conducted in the Ohio SLE Study (OSS). Although the model is simple, it nevertheless provides a useful first step in suggesting possible approaches to effective integration of LN biomarker data.

### Autoimmunity and inflammation

Although autoimmunity initiates SLE and subsequently LN, the molecular and cellular mechanisms that trigger this autoimmunity are not discussed here. For this work it is assumed that autoimmunity has already been initiated and the body's immune system has turned on itself to attack normal tissue. Helper T cells (Th2) produce cytokines (IL2, IL4, IL10) that help B cells proliferate and mature as auto-antibody producing cells. Released by the differentiated B cells into the blood, these auto-antibodies combine with self-antigens and form immune complexes. Under normal conditions, immune complexes are rapidly removed from the bloodstream and tissue by mechanisms involving the complement system, erythrocyte complement receptors, and phagocyte complement and Fc receptors [10,11]. During autoimmunity, however, the continuous production of auto-antibodies, in conjunction with defects in the clearance system, allows immune complexes to deposit in various organs, like the kidneys in LN. The localization of immune complexes in tissues is influenced by the nature of the antigen, the class of the antibody, and the size of the complex.

The complement system is part of the innate immune system, and consists of a group of soluble circulating proteins and cell-bound receptors. The complement system is activated by immune complexes, and as mentioned, is important for the proper clearance of immune complexes. However, when locally deposited immune complexes activate the complement system, the cascade of biochemical events results in the release of pro-inflammatory mediators that can increase vascular permeability, draw leukocytes to the area of immune complex localization, and directly induce tissue damage. Leukocytes are also activated by complement, and by direct interaction with antibodies in the immune complex via Fc receptors. This activation leads to more vascular damage and tissue destruction through the release of pro-inflammatory cytokines, toxic oxygen products, and proteolytic lysosomal enzymes. Coincident with these pro-inflammatory processes, anti-inflammatory mechanisms are activated to help control inflammation, however in LN these are generally overwhelmed. Prolonged inflammation is undesirable because it is characterized by healing of the tissue through scarring, causing the loss of normal tissue architecture. This can lead to chronic organ dysfunction.

### Therapy

Prognosis and outcome of LN can usually be improved dramatically by treatment. The considerations regarding the treatment of LN rest on an accurate assessment of the type and severity of renal involvement [4,5]. Current treatment for patients with severe kidney disease generally involves high dose corticosteroids accompanied by cytotoxic drugs that reduce the harmful effects of humoral or cellular immunity, and thereby allow the body to reestablish immunologic homeostasis.

The goal of treatment is to induce sustained remission, preserve renal parenchyma, and stabilize or improve kidney function (normalize serum creatinine). The time to reach remission varies from patient to patient, but early remission is a predictor of good prognosis. However, despite therapy, many patients flare again, raising questions about the effectiveness of immunosuppressive therapies, and the pathogenesis of LN flare. The efficacy of therapy may be dependent on when it is initiated relative to the status of renal injury, dosing of therapy, and drug combinations.

### Biomarkers/urine chemokines

To improve clinical treatment protocols, biomarkers that reflect different phases of the LN flare cycle have been sought in recent years. In this regard, we consider phases of a flare cycle as those times representing baseline, immediately before flare, at flare and immediately after flare. Most of these putative biomarkers are urine and serum factors closely related to renal flare cycles. One such group of biomarkers are the various complement proteins and activated fragments [12], though it is still unclear how clinically useful these are. Another candidate group of biomarkers are urine chemokines, which appear to change in amount with disease activity [9]. These chemotactic factors are believed to be induced locally within the kidney by the immune complex accumulation, and appear to be responsible for amplifying the inflammatory response by recruiting additional leukocytes to the kidney, thereby mediating tissue injury and renal dysfunction. The chemokine that has received the most attention in this regard is monocyte chemotactic protein-1 (MCP-1). Other potential urine biomarkers of LN activity include the iron regulatory hormone hepcidin, and the adipokine adiponectin [6-9].

### Modeling LN dynamics

The most frequent test ordered for the evaluation of LN activity is the urine protein level. Although proteinuria is an accepted LN clinical biomarker, it does not accurately forecast the LN flare cycle. Furthermore, while complement proteins, urine MCP-1 (uMCP-1), adiponectin, and hepcidin have been proposed as candidate LN flare cycle biomarkers, it is presently not clear how these would be used clinically to provide diagnostic, pathologic, or therapeutic information on each phase of the flare cycle to significantly impact LN treatment.

To accurately describe the complex dynamics of the renal flare, models incorporating these LN biomarkers need to be built to effectively capture the multiple time-dependent interactions among the biomarkers and other variables involved in the disease. Statistical models applied to large population clinical studies have been successful in highlighting relationships and correlations among various biological quantities, but have so far failed to provide reliable quantitative or even qualitative models [13].

Another way to address the issue of complex biological interactions and their effects is by means of mathematical modeling. Here we propose a mathematical model of LN dynamics based on a set of known biological interactions and experimental investigations. The model reproduces temporal changes in disease activity, including some LN urine biomarker profiles. We suggest that this model, paired with further clinical and experimental investigations, will provide a basis for more comprehensive modeling and improved clinical care for LN patients.

## Materials and methods

### Study data

**Figure 1:**
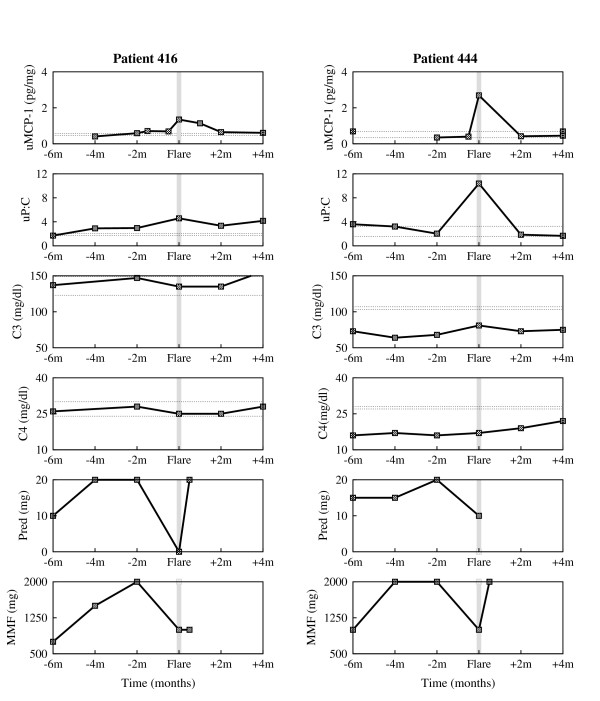
**Experimental data of individual patients enrolled in the Ohio SLE Study (I)**. Clinical measurements of urine MCP-1, urine P:C, serum C3 and serum C4 taken every 2 months, and accompanying therapy (Prednisone (Pred) = corticosteroids, Mycophenolate Mofetil (MMF) = immunosuppressants) around 6 months before flare and 4 months after flare, for patient 416 (first column) and patient 444 (second column). The horizontal dotted lines represent baseline values determined at two different time points that were at least 6 months from any flare activity. The gray vertical line marks the renal flare.

**Figure 2:**
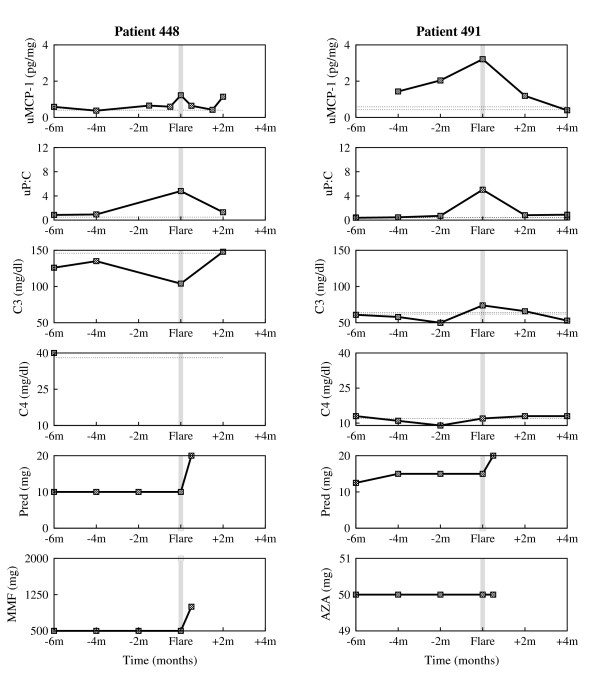
**Experimental data of individual patients enrolled in the Ohio SLE Study (II)**. Clinical measurements of urine MCP-1, urine P:C, serum C3 and serum C4 taken every 2 months, and accompanying therapy (Prednisone (Pred) = corticosteroids, Mycophenolate Mofetil (MMF), Azathioprine (AZA) = immunosuppressants) around 6 months before flare and 4 months after flare, for patient 448 (first column) and patient 491 (second column). The horizontal dotted lines represent baseline values determined at two different time points that were at least 6 months from any flare activity. The gray vertical line marks the renal flare.

The data examined here came from patients enrolled in the prospective longitudinal study OSS. Patients in OSS had four or more American College of Rheumatology criteria for SLE, and either currently active SLE, two or more SLE flares that required an increase in therapy in the preceding three years, or persistently active SLE defined as more than four months of activity despite therapy. Most patients were receiving maintenance immunosuppressive therapy before flare. Each patient was evaluated clinically and with laboratory tests every two months regardless of disease activity, and provided blood, a 24 hour urine specimen, and a freshly voided urine specimen at the visit. Renal and nonrenal flares were identified and uMCP-1, urine protein to urine creatinine ratio (uP:C), and plasma levels of complement components C3 and C4 were measured. Serial measurements from four individual patients, accompanied by therapy recordings when available, are shown in Fig. 1 and Fig. 2.

### Model description

We introduce here a model of kidney inflammation sustained by autoimmunity and damaged tissue. Based on the assumption that LN is mainly due to immune complex accumulation and resulting inflammation [3], the model captures the temporal behavior of serial measurements of candidate biomarkers from patients with unstable LN disease activity.

**Figure 3:**
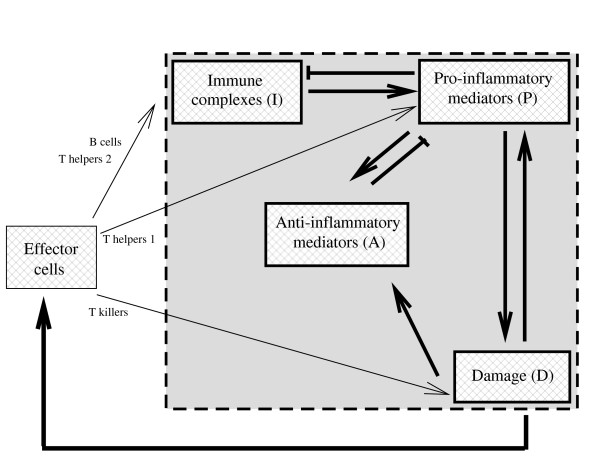
**Network of interactions that mediate renal damage in lupus nephritis**. Naive T cells (not shown) are activated by the self-antigen presenting cells (APCs), and release cytokines and various chemical signals that stimulate the activity of other immune cells, such as natural killer cells, helper T cells, B cells and macrophages. Each of these activation pathways can lead to tissue destruction. Frequently, helper T cells can cause local inflammation and tissue damage by recruiting macrophages via cytokines and chemokines. Tissue damage can also occur directly via the activity of cytotoxic natural killer cells. However, extensive tissue damage is due to auto-antibodies, produced by the B cells. These auto-antibodies form immune complexes with self-antigen, either by binding directly to cell surface antigens, or by forming immune complexes in the circulation that deposit in the kidney. Immune complexes activate the complement system (not shown), which recruits and activates effector leukocytes (e.g. neutrophils, macrophages). These pro-inflammatory activated leukocytes produce toxic products that damage tissue. Concurrent activation of anti-inflammatory cells and production of anti-inflammatory mediators counterbalance the action of pro-inflammatory mediators. The flare process undergoes positive feedback because debris from apoptotic and damaged cells further stimulates the autoimmune response. As the flare is treated, activated effector cells are reduced, the production of auto-antibodies is disrupted, the deposition of immune complexes decreases, and tissue that is not permanently scarred undergoes repair or regeneration. Our mathematical model, Eqs (1)-(4), builds on the gray box interactions and follows the evolution in time of four variables: immune complexes (*I*), pro-inflammatory mediators (*P*), damaged tissue (*D*), and anti-inflammatory mediators (*A*).

Fig. 3 summarizes the mechanisms upon which our model is built. The schematic diagram represents a network of interactions that mediate renal damage in LN. Naive T cells (not shown) are activated by the self-antigen presenting cells (APCs), and release cytokines and various chemical signals that stimulate the activity of other immune cells, such as natural killer cells, helper T cells, B cells and macrophages. Each of these activation pathways can lead to tissue destruction. Frequently, helper T cells can cause local inflammation and tissue damage by recruiting macrophages via cytokines and chemokines. Tissue damage can also occur directly via the activity of cytotoxic natural killer cells. However, the most extensive tissue damage is due to auto-antibodies, produced by the B cells. These auto-antibodies form immune complexes with self-antigen, either by binding directly to cell surface self-antigens, or by forming immune complexes in the circulation that get deposited in the kidney. Immune complexes activate the complement system (not shown), which recruits and activates effector leukocytes (e.g. neutrophils, macrophages). These pro-inflammatory activated leukocytes produce toxic products that damage tissue. Concurrent production of anti-inflammatory cells and chemicals counterbalance the action of pro-inflammatory mediators. The flare process undergoes positive feedback because debris from apoptotic damaged cells further stimulates the autoimmune response. As the flare is treated, activated effector cells are reduced, the production of auto-antibodies is disrupted, the deposition of immune complexes decreases, inflammation is resolved, and tissue that is not permanently scarred undergoes repair or regeneration.

Because LN develops in parallel with the systemic disease of SLE, it is hard to draw distinction between clinical manifestations that are only relevant to LN. While we cannot ignore the contribution of systemic disease to temporal changes of the LN biomarkers, some LN biomarkers, such as uMCP-1, appear to be specific and do not reflect systemic disease activity.

Of all the paths leading to renal dysfunction in SLE, we have assumed that immune complex-mediated damage is central to LN. This simplified view of the interactions relevant to lupus renal flares is shown in the gray background area of Fig. 3. The simplified model does not address the spatial, dynamic, and compartmental aspects (blood, tissue, etc.) of the immune and inflammatory responses.

### Model variables

The mathematical model builds on the gray box interactions and follows the evolution in time of four variables:

• *Immune complexes (I)*, implicitly related to other components of the immune system which contribute to the formation of immune complexes (antigens, antigen presenting cells, T cells, B cells);

• *Pro-inflammatory mediators (P)*, that represent the combined effect of immune cells such as macrophages and lymphocytes, and pro-inflammatory mediators, such as complement (as measured by C4 or C3), MCP-1, TNF-*α*, IL-1-*β*;

• *Damaged tissue (D)*, namely, healthy tissue that has been damaged by the immune cells and/or immune complexes, and is undergoing apoptosis or necrosis;

• *Anti-inflammatory mediators (A)*, that represent the combined effect of anti-inflammatory cells, anti-inflammatory cytokines such as IL-10, TGF-*β*, as well as therapeutics.

### Model equations

#### Equation for I (immune complexes)

**Figure 4:**
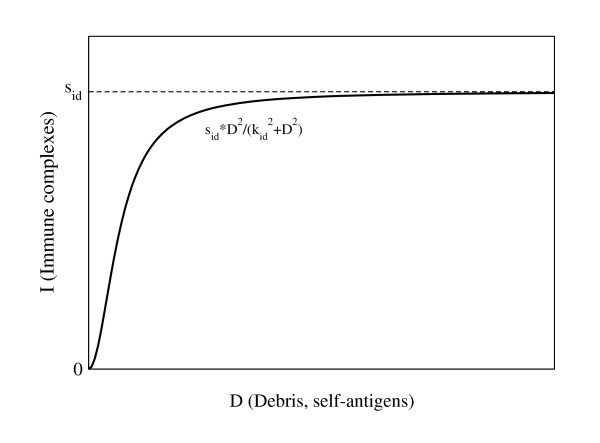
**Hill functional of order 2**. We represent the immune complexes (*I*) formation due to accumulation of self-antigens from debris *D*, by a Hill functional of order 2, . When there are only a few antigens around, not many immune complexes are produced; as antigens accumulate, more immune complexes are being created, and saturation, *s*_*id*_, is reached for sufficiently many self-antigens.

The model assumes that circulating immune complexes deposit in the kidneys at a rate *s*_*i*_. This term is also a base value for the activity of the complement system. Although complement activation in the tissue and at the site of tissue damage will occur under at least three scenarios when considering SLE (when *I *form in the circulation, when *I *deposit in tissue, and when tissue damage occurs), we average them here for simplicity. Apart from the immune complexes passively trapped within glomeruli, we also account for immune complexes formed as a result of self-antigen accumulation within the tissue. A reasonable function for the *I *inducement is considered to be a sigmoid (S-shape) function as shown in Fig. 4. Thus, as in [14-16], we take here a functional response of Hill kinetics of order 2, assuming that just a few self-antigens will not raise a strong immune response, but as debris accumulates the immune response is gradually induced, and saturation, *s*_*id*_, is reached for sufficiently many self-antigens. The accumulation of immune complexes activates the complement cascade, generating peptides and chemotactic factors that trigger the inflammatory response, with various mediators being activated and cells being recruited (at rate *k*_*pi*_) to remove the immune complexes from the system (at rate *k*_*ip*_). In summary,(1)

Here and in the following, for simplicity, we take all the functions *f *to be the same, but they will also depend on the anti-inflammatory mediators; see Eq. (5).

#### Equation for P (pro-inflammatory mediators)

The prolonged presence of immune complexes sets the stage for more damaging inflammatory events. The immune response is amplified by existing immune cells and pro-inflammatory mediators, providing positive feedback at rate *k*_*pi*_, respectively *k*_*pp*_. To these immune responses, we add a term that accounts for the activation of pro-inflammatory agents as a result of cytokines released or induced by damaged tissue, at rate *k*_*pd*_. This term accounts for the clinically observed increase in the number of immune cells in the kidney due to infiltration by circulating leukocytes. As the infiltration in a non-lymphoid organ is usually due to biologic mediators released by damaged cells themselves and/or by resident or infiltrated leukocytes stimulated by the damaged cells, the infiltration term is taken to be dependent on the concentration of damaged cells; this also ensures that in the absence of damaged cells there is no infiltration. By including decay of pro-inflammatory mediators at rate *μ*_*p*_, we have(2)

#### Equation for D (damaged tissue)

The damaged tissue not only releases pro-inflammatory cytokines (at rate *k*_*pd*_) that cause further immune cells activation, but also the phagocytosis of immune complexes by immune cells can result in release of cytokines and toxins that lead to tissue damage [17,18], a phenomenon described here by the first term in the equation for *D*. The positive feedback interactions between immune cells and damage exists even in the absence of immune complexes and can be triggered by other stimuli, such as tissue trauma [19]. We take *k*_*dp *_the rate at which collateral damage is produced by the pro-inflammatory mediators. The decay rate of damage, *μ*_*d*_, is a combination of repair, resolution, and regeneration of tissue. Hence,(3)

#### Equation for A (anti-inflammatory mediators)

To keep the inflammation under control, most LN patients are regularly prescribed anti-inflammatory drugs. The anti-inflammatory therapy is mathematically modeled here by adding a source term *s*_*a *_in the equation for *A*. There is also intrarenal production of anti-inflammatory mediators, production correlated to the level of inflammation and damage, at rates *k*_*ap*_, and respectively *k*_*ad*_. Once activated, the anti-inflammatory chemicals inhibit the production of more pro-inflammatory mediators, decrease the ability of pro-inflammatory chemicals and cells to fight against immune cells, and lower the damage created by the inflammation. Unfortunately, the anti-inflammatory cytokines discordantly counter the effects of pro-inflammatory mediators, thus losing the battle. The use of immunosuppressive drugs allows some attenuation of the inflammation, so the natural anti-inflammation can be effective. Finally, the anti-inflammatory agents degrade at rate *μ*_*a*_. In summary,(4)

While directly lowered by the immunosuppression, both *s*_*i *_and *s*_*id*_, are also controlled by the endogenous anti-inflammatories. All these inhibitions are incorporated into the model by taking(5)

The functions *f *in the above equations need not all be the same, although they should have similar form and profile as the function in Eq. (5). However, in the absence of data, for simplicity, we have taken all these functions to be the same.

### Clinical relevance

In order to assess whether the model we developed here can be used to further study the dynamics of the disease, we compare the simulations of the model with clinical data presented in Fig. 1 and Fig. 2. In doing so, the surrogate marker for *P *will be the chemotactic factor MCP-1, represented here by the uMCP-1, which is thought to be mainly induced by the presence of the immune complexes. MCP-1 is a chemokine responsible for recruiting inflammatory cells to the kidney and activating these cells.

Blood or protein in the urine is a sign of kidney damage, as most proteins are too big to pass through the renal filtration barrier into the urine unless the glomeruli are damaged. Generally, worsening of proteinuria reflects the extent of kidney damage. Consequently, proteinuria, represented here by the uP:C, is taken as a surrogate clinical marker for acute kidney damage, *D*.

In addition to using urine biomarkers data when evaluating the efficacy of the model, therapy protocols are also considered when available. In the model, immunosuppression is enhanced due to any drug/event leading to decreased production of immune complexes. Therefore, in terms of model parameters, immunosuppressive therapy means decreasing the rate of immune complex deposition into the kidney, *s*_*i*_, and/or decreasing the rate of intrarenal production of immune complexes, *s*_*id*_. In LN either steroids or immunosuppressants can trigger these salutary effects. Lastly, the anti-inflammatory therapy is simulated as any drug/event leading to an increase of anti-inflammatory mediators, modeled here by the source term *s*_*a*_.

### Parameters

To explore the model computationally, the ordinary differential equations (1)-(4) are numerically solved in Matlab. Initial conditions are chosen to match the clinical data when available (*P*_0_, *D*_0_), and to depict some preexisting conditions stemming from earlier stages of the disease dynamics (*I*_0 _*> *0, *A*_0 _*> *0).

For each individual patient, not only we match *P*_0 _and *D*_0 _to uMCP-1 and uP:C measurements at 6 months before flare, but we also estimate patient-specific parameters that would produce results consistent with individual biological observations on these urine biomarkers. In that sense, solutions to the differential equations for *P *and *D *fit the data sets on uMCP-1 and uP:C, respectively, for each of the four patients considered.

In fitting the model to clinical data, we fixed *μ*_*a *_to a biologically realistic value. Based on parameter values used in [19], the decay rate of the anti-inflammatory mediators is calculated using an average half-life of 7.5 h (≈ 0.31 days).

Since the model variables represent various types of cells and cytokines concurrently, units for the model variables are not fixed, but rather represented as generic units like *I*-units, *P*-units, *D*-units, and *A*-units. However, as we are interested in possible biological and medical applications of the mathematical model, we compare some of the generalized variables with clinically-measured variables (like uMCP1 and uP:C), and calibrate the model to these available clinical data.

## Results

We incorporate clinical measurements of uMCP-1 and uP:C from OSS LN biomarker discovery studies into the mathematical model, to find sets of parameters that best describe data and disease dynamics. Estimated parameter values are then used to perform computational experiments that address model usefulness. We show that effective combination of clinical data and mathematical modeling can improve our understanding of disease dynamics, and can be used to gain insight into why failures occur with the way LN is currently treated.

### Comparison of simulated uMCP-1 and uP:C dynamics during flare cycle with patients data

As disease progression and response to treatment varies from one patient to another, we choose to separately calibrate the mathematical model to each individual patient clinical data shown in Fig. 1 and Fig. 2. More precisely, we fit the clinical urine biomarkers data sets on uMCP-1 and uP:C to the solutions of the differential equations for *P *and *D*, respectively, to derive the patient-specific parameter estimates listed in Table 1 and Table 2. The derivations were handled with the curve fitting tool of Berkeley Madonna [20], using nonlinear least-squares regression that minimizes the sum of the squared residuals between the clinical data and the computational solutions.

**Table 1:** Initial conditions and parameter estimates that correspond to the best fit of the model to the data.

	Patient 416	Patient 444	Patient 448	Patient 491	Units
*I*_0_	0.1	0.1	0.1	0.1	*I*-units
*P*_0_	0.4	0.5	0.58	0.5	*P*-units
*D*_0_	1.7	3.59	0.85	0.38	*D*-units
*A*_0_	0.1	0.1	0.1	0.1	*A*-units
*s*_*i*_	0.001-0.005	0.5-6	0.02-4.5	0.1-0.5	*I*-units day^-1^
*s*_*id*_	0.002-0.015	0.5-2	0.02-3.8	0.1-0.6	*I*-units day^-1^
*k*_*id*_	1	1	1	1	*D*-units
*k*_*ip*_	0.025	0.015	0.01	0.003	*P*-units^-1 ^day^-1^
*k*_*pi*_	0.13	0.01	0.006	0.05	*P*-units *C*-units^-1 ^day^-1^
*k*_*pp*_	0.02	0.015	0.02	0.12	day^-1^
*k*_*pd*_	0.001	0.001	0.001	0.01	*P*-units *D*-units^-1 ^day^-1^
*μ*_*p*_	0.06	0.06	0.13	0.33	day^-1^
*k*_*dip*_	0.025	0.015	0.01	0.003	*P*-units^-1 ^day^-1^
*k*_*dp*_	0.27	0.01	0.03	0.015	*D*-units *P*-units^-1 ^day^-1^
*μ*_*d*_	0.04	0.015	0.03	0.035	day^-1^
*s*_*a*_	0.05-0.3	2-7	0.4-10	1-4	*A*-units day^-1^
*k*_*ap*_	0.022	0.006	0.035	0.001	*A*-units *P*-units^-1 ^day^-1^
*k*_*ad*_	0.22	0.06	0.35	0.01	*A*-units *D*-units^-1 ^day^-1^
*μ*_*a*_	2.2	2.2	2.2	2.2	day^-1^
*A*_*inf*_	0.45	0.45	0.45	0.45	*A*-units

For each patient, parameter estimates are given along with the initial conditions for the best fits of the *P *and *D *model solutions to the serial measurements of uMCP-1 and uP:C, respectively. Detailed information on parameter estimates for *s*_*i*_, *s*_*id*_, and *s*_*a *_is included in Table 2.

**Table 2:** Parameter estimates for *s*_*i*_, *s*_*id*_, and *s*_*a*_, that correspond to the best fit of the model to the data.

	Patient 416			Patient 444			Patient 448			Patient 491		
Time frame	*s*_*i*_	*s*_*id*_	*s*_*a*_	*s*_*i*_	*s*_*id*_	*s*_*a*_	*s*_*i*_	*s*_*id*_	*s*_*a*_	*s*_*i*_	*s*_*id*_	*s*_*a*_
[-6*m*, -4*m*]	0.002	0.005	0.05	6	1	3	0.2	0.26	0.4	0.5	0.6	1
[-4*m*, -2*m*]	0.001	0.003	0.1	2	0.5	4	0.2	0.26	0.4	0.5	0.6	1.2
[-2*m*, -2*w*]	0.005	0.015	0.05	2	0.5	2	0.02	0.06	0.4	0.5	0.6	1.2
[-2*w, Flare*]	0.005	0.015	0.05	5	2	2	4.5	3.8	0.4	0.5	0.6	1.2
[*Flare*, +2*w*]	0.001	0.002	0.3	0.5	0.5	7	0.06	0.02	5	0.1	0.1	2.4
[+2*w*, +6*w*]	0.001	0.002	0.3	0.5	0.5	7	0.06	0.02	10	0.1	0.1	2.4
[+6*w*, +2*m*]	0.001	0.002	0.3	0.5	0.5	7	0.06	0.02	2	0.1	0.1	2.4
[+2*m*, +4*m*]	0.005	0.012	0.1	1.5	0.5	7	0.06	0.02	2	0.1	0.1	4

Because parameters *s*_*i*_, *s*_*id *_and *s*_*a *_are used to reflect therapy changes and/or therapy effects on the disease dynamics, they are in general time dependent. All these parameters may vary greatly during the flare cycle (changes in therapy, therapy failure or success), and can also vary greatly from patient to patient (stages of disease, general patient health).

**Figure 5:**
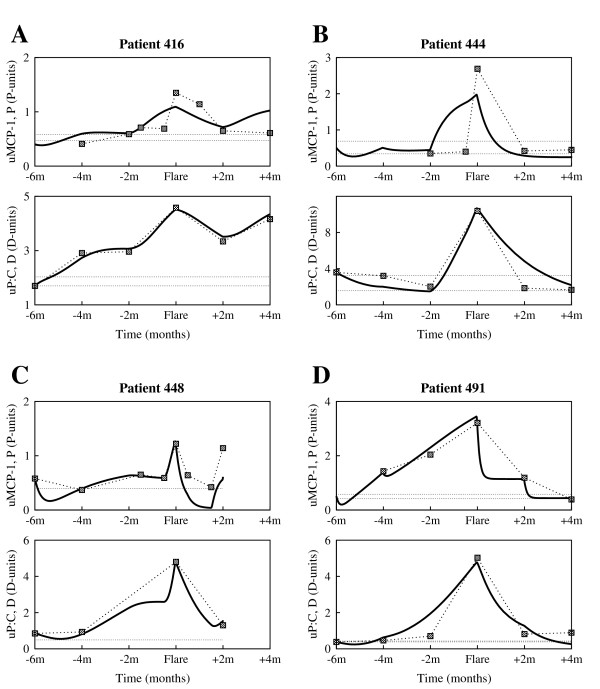
**Fit of the model (solid curves) to experimental data (filled squares) collected from patients enrolled in the Ohio SLE Study**. **(A) **Patient 416; **(B) **Patient 444; **(C) **Patient 448; **(D) **Patient 491. Each pair of graphs presents the pro-inflammatory mediators (*P*) (top panels, solid curve), and the tissue damage (*D*) (bottom panels, solid curve) dynamics during flare cycle (6 months before and 4 months after the renal flare), against the patient data (filled squares) on uMCP-1 and uP:C, respectively. The horizontal dotted lines mark the baseline values determined at two different time points that were at least 6 months from any flare activity. The solid curves are obtained by numerically solving Eqs. (2) and (3) with the parameters listed in Table 1 and Table 2. Parameter estimations were performed with the curve-fitting tool of Berkeley Madonna [20]. With few exceptions, the model simulations reproduce the experimental measurements around the renal flare. The difficulty in exactly matching the simulations to the experimental data is due to the simplifications assumed when constructing the model; for example *P *reflects the overall activity of all pro-inflammatory mediators, rather then the uMCP-1 dynamics alone.

Comparisons of model simulations to clinical data are shown in Fig. 5, panels A-D (solid curves). The filled squares connected by the dotted curves represent the clinical serial measurements of uMCP-1 and uP:C, taken approximately two months apart. The flare cycle spans ten months, six months before flare to four months after the renal flare. We have found that the model qualitatively and (to some degree) quantitatively reproduces the experimental measurements around the renal flare. Given the simplifications imposed on the model we expect some variations from data. For example, in the model *P *reflects the overall activity of all pro-inflammatory mediators, rather then the uMCP-1 dynamics alone.

Because parameters *s*_*i*_, *s*_*id *_and *s*_*a *_are used to reflect therapy changes and/or therapy effects on the disease dynamics, these parameters are in general time dependent; *s*_*i*_, for instance, may vary greatly during the flare cycle (changes in therapy, therapy failure or success), and can also vary greatly from patient to patient (stages of disease, general patient health). Taking them as piecewise constant is just a way of simplifying the computations, but one may think of the solutions as smooth functions which are approximated by the discontinuous ones.

Although the Berkeley Madonna software finds parameter estimates that correspond to the best fit of the model to the data, there is some variance which of course increases if the data set is too small. We acknowledge that our data set is small, but this is the state of the experimental knowledge at this time. Findings from clinical studies with more frequent sampling, as well as incorporating more detailed biological information, would be useful to improve the accuracy of parameter estimation and data fitting.

### The parameterized model can be used to gain insight into disease mechanisms

Parameter values that best describe the data could yield valuable insights into the dynamics of pro- and anti-inflammatory pathways within the kidneys during LN. For example, intrarenal levels of immune complexes could be modulated by increasing the rate at which the immune complex deposit into the kidney (*s*_*i*_), or by increasing the immune response to accumulation of damaged cells (*s*_*id*_). In spite of the fact that both mechanisms are targeted by maintenance immunosuppressive therapy, during disease flare new immune complexes continue to add to the initial levels and aggravate the symptoms. For patients considered here, major changes in disease dynamics are mostly explained by significantly increased intrarenal immune complexes levels beginning 2 months to 2 weeks before flare (patients 416, 444 and 448), as suggested by the estimated values of *s*_*i *_and *s*_*id*_. The values for *s*_*a*_, and the information related to ongoing therapy for each individual patient, suggest that immune complexes levels increase when therapy is too weak (patients 448 and 419) or timed incorrectly (patients 416 and 444). Another reason for the sudden increase of *s*_*i *_and *s*_*id *_while on therapy could be that during immunosuppression a higher amount of self-antigen from the apoptotic cells is needed to activate the immune cells to produce auto-antibodies. Once that threshold is reached maintenance therapy cannot control the immune response wave. Unsuccessful therapy could also theoretically be explained by a shorter half-life of endogenous anti-inflammatory factors compared to the half-lives of pro-inflammatory mediators and damaged tissue.

### The parameterized model can be used to explore therapeutic strategies

We are interested not only in matching model simulations with clinical data and learning about possible mechanisms leading to renal flare, but also in investigating therapeutic implications of these results. By computational experiments in which inputs and parameters are varied, we explored the responses of the model to various clinical interventions, and identified administration strategies that would result in the best outcomes. This is illustrated here for each of our four patients individually, but the method can be applied to any group of patients that have similar clinical manifestations.

#### Patient 416

**Figure 6:**
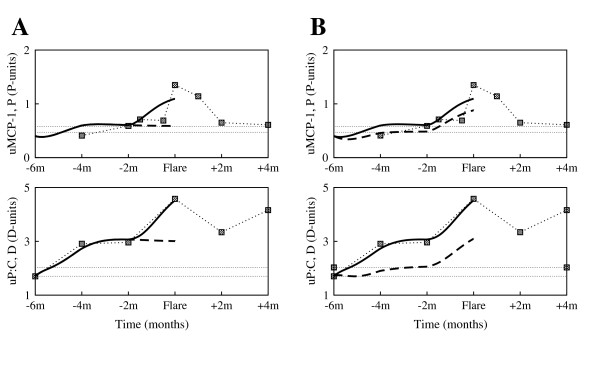
**Before flare interventions: simulations for patient 416**. **(A) **Instead of decreasing the dose of corticosteroids and immunosuppressants at 2 months before flare (as shown in Fig. 1), maintaining the therapy dosage lowers the levels of both pro-inflammation and damage; *s*_*i *_= 0.005 *I*-units day^-1^, *s*_*id *_= 0.015 *I*-units day^-1^, *s*_*a *_= 0.05 *A*-units/day, at 2 months before flare (solid curves); *s*_*i *_= 0.001 *I*-units day^-1^, *s*_*id *_= 0.003 *I*-units day^-1^, *s*_*a *_= 0.1 *A*-units/day, at 2 months before flare (dashed curves). **(B) **Increasing the half-life of the anti-inflammatory mediators by decreasing the decay rate *μ*_*a*_, also controls the inflammation wave; *μ*_*a *_= 2.2/day (solid curves); *μ*_*a *_= 1.1/day (dashed curves). All other parameters are as listed in Table 1 and Table 2.

Despite baseline complement component levels, and increased immunosuppression, there is worsening of symptoms leading to flare. One can argue that renal flare is a result of either inadequate handling of immunosuppression at 2 months before flare, or low anti-inflammatory levels (*s*_*a *_= 0.05 *A*-units/day). Fig. 6A shows that pro-inflammation and organ dysfunction could be significantly lowered by maintaining the therapy at the 4 months before flare levels (dashed curves). To address the low levels of anti-inflammation, one could increase the anti-inflammatory therapy at any time before flare, by increasing *s*_*a *_(results not shown). Alternatively, methods that increase the half-life of anti-inflammatory mediators, i.e. from 0.3 days to 0.6 days, would also successfully lower inflammation and reduce tissue damage (Fig. 6B, dashed curves).

### Patient 444

**Figure 7:**
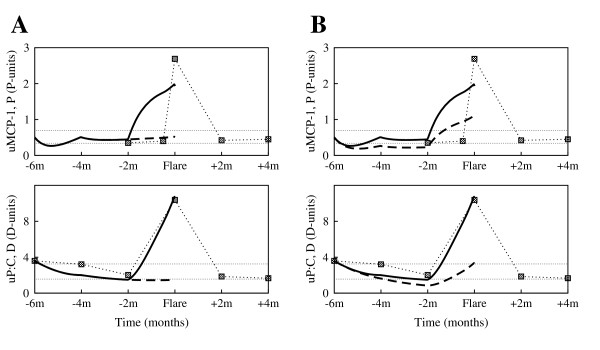
**Before flare interventions: simulations for patient 444**. **(A) **No drop in the anti-inflammatory therapy 2 months before flare would keep pro-inflammation and damage low; *s*_*a *_= 2 *A*-units/day, 2 months before flare (solid curves); *s*_*a *_= 4 *A*-units/day, 2 months before flare (dashed curves). **(B) **Therapeutic strategies aimed at restricting immune complex infiltration into the kidneys and the production of more immune complexes as a result of debris accumulation, could also improve disease symptoms; *s*_*i *_= 6 *I*-units/days, and *s*_*id *_= 1 *I*-units/days (solid curves); *s*_*i *_= 3 *I*-units/days, and *s*_*id *_= 0.5 *I*-units/days (dashed curves). All other parameters are as listed in Table 1 and Table 2.

Low complement component values suggest a significant increase of intrarenal immune complexes, even as immunosuppression dose is increased 4 months before flare. While the high levels of pro-inflammatory agents alone would explain the renal flare, the drop in therapy 2 months before flare could also be responsible. If the anti-inflammatory therapy would have been kept stronger, *s*_*a *_= 4, 2 months before flare (Fig. 7A, dashed curves), the clinical outcome would have been greatly improved. Moreover, if the anti-inflammatory drugs would have been stronger immuno-inhibitors and lower *s*_*i *_and *s*_*id *_even more, no change in anti-inflammation would have been needed to improve disease dynamics (Fig. 7B, dashed curves). Given that immune complexes are a leading cause of damage and their numbers are not lowered by current therapy, we could also explore other ways of reducing the level of immune complexes. One option is to increase the rate at which phagocytes remove immune complexes from the system, *k*_*ip*_. However that could inadvertently increase tissue damage by the activated phagocytes, and make more debris available to trigger increased production of immune complexes (results not shown). Decreasing the half-life of damaged tissues - i.e. by facilitating apoptosis, or the clearance of the damaged cells, could be a better option, as will be illustrated for the next patient.

#### Patient 448

**Figure 8:**
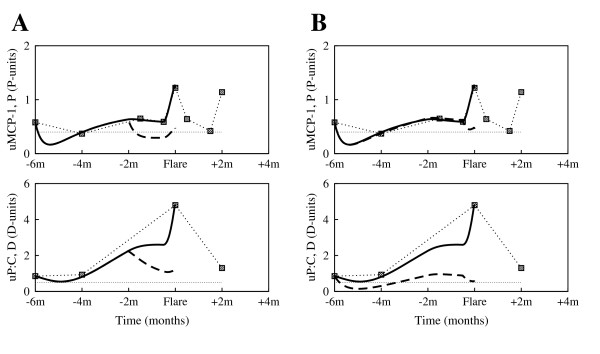
**Before flare interventions: simulations for patient 448**. **(A) **Worsening of LN symptoms is greatly improved if the maintenance therapy is accompanied by an increase in anti-inflammatory therapy 2 months before flare, with close results when the decision is delayed for 6 more weeks (results not shown); *s*_*a *_= 0.4 *A*-units/day, 2 months before flare (solid curves); *s*_*a *_= 1.5 *A*-units/day, 2 months before flare (dashed curves). **(B) **When the increase of the anti-inflammatory treatment is delayed 2 weeks before flare, parallel therapeutic strategies aimed at faster removal of apoptotic cells greatly improves the outcome;, *μ*_*d *_= 0.03/day, and *s*_*a *_= 0.4 *A*-units/day at 2 weeks before flare (solid curves); *μ*_*d *_= 0.1/day, and *s*_*a *_= 1.5 *A*-units/day at 2 weeks before flare (dashed curves). All other parameters are as listed in Table 1 and Table 2.

Maintenance therapy did not avoid LN symptoms. We therefore compare outcomes of a stronger therapy, increasing the anti-inflammatory drug dose, either at 2 months before flare (Fig. 8A, dashed curves), or later at 2 weeks before flare (results not shown). In both cases, pro-inflammation and tissue dysfunction tend to improve rapidly. Whereas pro-inflammation is low with or without intervention, timing of intervention affects damage decrease. Impaired clearance of apoptotic cells could explain the elevated levels of damage vs. lower levels of pro-inflammation. Even a later increase of corticosteroids, at 2 weeks before flare, results in a lower range of damage and dysfunction, when the removal rate of damage cells is increased (Fig. 8B, dashed curves). It is reasonable to assume that strategies to lower the formation of apoptotic cells could be helpful in reducing the intensity of LN symptoms.

#### Patient 491

**Figure 9:**
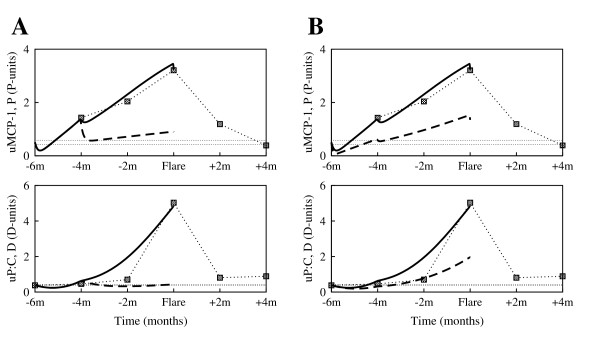
**Before flare interventions: simulations for patient 491**. **(A) **A larger increase of the corticosteroid dose 4 months before flare would control the inflammation and avoid clinical manifestations of the renal flare; *s*_*a *_= 1.2 *A*-units/day 4 months before flare (solid curves); *s*_*a *_= 2 *A*-units/day 4 months before flare (dashed curves). **(B) **The initial anti-inflammatory therapy would have better results if methods to lower the half-life of the pro-inflammatory mediators, *μ*_*p*_, could also be implemented; *μ*_*p *_= 0.33/day (solid curves), and *μ*_*p *_= 0.66/day (dashed curves). All other parameters are as listed in Table 1 and Table 2.

Both, uMCP-1 and uP:C, levels are above the baseline during the whole flare cycle (Fig. 9, solid curves). But, as shown in Fig. 9A (dashed curves), increased corticosteroid dose 4 months before flare would control the inflammation and avoid clinical manifestations of the renal flare. The significant increase of pro-inflammation could also be explained by half-life differences, with the pro-inflammatory mediators having a much longer half-life than the anti-inflammatory mediators. The initial anti-inflammatory therapy would have better results if methods to lower the half-life of the pro-inflammatory mediators, *μ*_*p*_, would have been also implemented (Fig. 9B, dashed curves).

## Conclusions

Lupus nephritis is a chronic, relapsing-remitting autoimmune disease that damages the kidneys. It is caused by immune complex/auto-antibody accumulation within the kidneys, resulting in inflammatory injury to the kidneys. Left untreated, LN causes kidney failure that may necessitate dialysis or a kidney transplant. High doses of steroids and immunosuppressive drugs, that globally suppress the immune system, are used in patients with severe LN. Unfortunately, despite aggressive therapy only about 50% of LN patients experience a complete or partial response by 6 months.

Reasons for unsuccessful treatments include the complexity of the disease itself, the lack of reliable outcome measures, a limited understanding of the pathogenesis of the disease, the heterogeneity of the patient population, the unpredictable course of disease in individual patients, and the lack of reliable biomarkers [21]. Consequently, to improve LN outcome it is necessary to identify clinically relevant biomarkers that can provide diagnostic, pathogenic and therapeutic information on each phase of the flare cycle. Moreover, to accurately follow disease progression and impact the outcome, such biomarkers need to be integrated into global LN-specific models of autoimmunity and inflammation. These models can exhibit various outcomes and facilitate the understanding of the complex interactions relevant to LN, adding to the statistical efforts that seek correlations among various clinical data and outcomes in large patient populations.

### Summary of paper

This is the first mathematical model that describes the chronic disease of LN in terms of a dynamic system. Based on differential equations that describe the dynamics of immune cells, pro- and anti-inflammatory mediators, and global tissue damage/dysfunction, this model represents, in a simplified way, the complexity of interactions underlying disease activity. Despite its simplicity, when calibrated with actual individual patient data sets, the model qualitatively reproduces the observed clinical behavior for each patient, and can be used to better understand disease mechanisms specific to each patient. Furthermore, by computational experiments in which inputs and parameters are varied, the model provides a framework for future modeling opportunities related to LN. Specifically, the model may be used to evaluate therapeutic strategies for individual patients, or a group of patients that share similar data patterns. Additionally, this may be a feasible approach to personalizing care for LN patients.

### Model usefulness

Our computational experiments reproduce many observations characteristic of LN disease progression, and support the hypothesis that inflammation plays an important role in LN dynamics and flare severity. In light of our simulations, it is reasonable to assume that therapies aimed at controlling inflammation and/or balancing the activity of anti-and pro-inflammatory mediators could improve the treatment outcome for LN patients. Furthermore, extended versions of the model could be used to computationally test the outcome of manipulating the chemokine network in experimental murine models of SLE nephritis [9], and simulations may also point to novel potential biomarkers to evaluate disease mechanisms and the response to therapy.

We have also shown with this model, in a number of clinical cases, how continuous deposition of immune complexes into the kidney can lead to persistent inflammation and subsequent tissue damage. This suggests that successful therapies may be aimed at controlling both immune complex formation/deposition, and the pro-inflammatory response. Timing and combination of various therapies may be important, and models like ours are cost-efficient ways to test different protocols in a timely manner.

Rather than predicting a flare, we suggest that close monitoring of biomarkers such as uMCP-1 and uP:C, in conjunction with the mathematical model, can provide pathogenic (disease evolution) and therapeutic (response to treatment) information that can be used to further guide interventions to decrease inflammation and tissue damage before more harm occurs. If a flare cannot be avoided, the model calibrated to pre-flare patient data could be used to learn particulars about disease dynamics, point towards causes and solutions, test possible therapies and compare outcomes, improve and shorten treatment decision making process. As individual flares are different, information from previous flares may not be very useful to treat future flares. Fortunately, our mechanistic mathematical model is particularly useful in such cases, as model equations (interactions) do not change, only model parameters (strength of interactions) vary to reflect new data (evolution of the disease and therapy). Maybe most important, the way parameters are estimated (i.e. for each set of data we choose the set of parameters for which simulations are closest to that clinical data), makes the model equally applicable to analyzing individual patient data, as well as data collected from a larger patient population. Although our goal is to identify strategies that would result in the best outcome for each individual patient, optimizing clinical trial design (e.g. identifying administration strategies for the entire cohort, help with patient selection given a treatment administration regimen) constitutes another potential application of our simulations.

### Model extensions

While we may not have captured all parameters accurately, the assumptions on which the model is based are biologically reasonable, and the results are a good fit with clinical data. However, in this first, simplified model, other relevant components of the immune system (antigens, antigen presenting cells, T cells, B cells) and inflammatory system (pro-inflammatory mediators like serum MCP-1, TNF-*α*, IL-1, and anti-inflammatory mediators like IL-10, TGF-*β*) are not included. In future studies, an extended model would include details on the activation of T cells by APC, recruitment of phagocytes and activation and regulation of the complement system. Future refinements of the model could also consider relations balancing anti-inflammatory vs. pro-inflammatory mediators (cells, cytokines) and the importance of these interactions in defining the disease dynamics (remission, flare). Drugs could also be added as separate variables to the model, and time-dependent protocols for administering the drugs could be explored.

## Competing interests

The authors declare that they have no competing interests.

## Authors' contributions

PBG, RCS and AF formulated the model equations and wrote the manuscript. PBG performed the numerical calculations. DJB and BHR were consulted on the model during the preparation of the paper, and all authors read and approved the manuscript.
